# Notch‐Expanded Murine Hematopoietic Stem and Progenitor Cells Mitigate Death from Lethal Radiation and Convey Immune Tolerance in Mismatched Recipients

**DOI:** 10.5966/sctm.2016-0112

**Published:** 2016-09-13

**Authors:** Filippo Milano, Fabiola Merriam, Ian Nicoud, Jianqiang Li, Ted A. Gooley, Shelly Heimfeld, Suzan Imren, Colleen Delaney

**Affiliations:** ^1^ Clinical Research Division, Fred Hutchinson Cancer Research Center, Seattle, Washington, USA; ^2^ Department of Medicine, University of Washington, Seattle, Washington, USA; ^3^ Department of Pediatrics, University of Washington, Seattle, Washington, USA

**Keywords:** Notch expansion, Hematopoietic‐acute radiation syndrome, Mismatched mouse progenitor cells, Skin grafts, Tolerance induction

## Abstract

The hematopoietic syndrome of acute radiation syndrome (h‐ARS) is characterized by severe bone marrow aplasia, resulting in a significant risk for bleeding, infections, and death. To date, clinical management of h‐ARS is limited to supportive care dictated by the level of radiation exposure, with a high incidence of mortality in those exposed to high radiation doses. The ideal therapeutic agent would be an immediately available, easily distributable single‐agent therapy capable of rapid in vivo hematopoietic reconstitution until recovery of autologous hematopoiesis occurs. Using a murine model of h‐ARS, we herein demonstrate that infusion of ex vivo expanded murine hematopoietic stem and progenitor cells (HSPCs) into major histocompatibility complex mismatched recipient mice exposed to a lethal dose of ionizing radiation (IR) led to rapid myeloid recovery and improved survival. Survival benefit was significant in a dose‐dependent manner even when infusion of the expanded cell therapy was delayed 3 days after lethal IR exposure. Most surviving mice (80%) demonstrated long‐term in vivo persistence of donor T cells at low levels, and none had evidence of graft versus host disease. Furthermore, survival of donor‐derived skin grafts was significantly prolonged in recipients rescued from h‐ARS by infusion of the mismatched expanded cell product. These findings provide evidence that ex vivo expanded mismatched HSPCs can provide rapid, high‐level hematopoietic reconstitution, mitigate IR‐induced mortality, and convey donor‐specific immune tolerance in a murine h‐ARS model. Stem Cells Translational Medicine *2017;6:566–575*

**Significance Statement**

There is an urgent need to develop a therapeutic agent for the treatment or supportive care of the hematopoietic syndrome of acute radiation syndrome (h‐ARS). This study reports on the infusion of mismatched cryopreserved ex vivo mouse hematopoietic stem and progenitor cells (HSPCs), expanded in the presence of Notch ligand, after a lethal dose of ionizing radiation (IR). The results were rapid donor engraftment, mitigated IR‐induced toxicity, and improved survival in a dose‐dependent manner. Moreover, these cells induced donor‐specific immune tolerance, resulting in longer survival of donor skin allografts. These findings reinforce that cord blood‐derived HSPCs, expanded ex vivo by using Notch ligand, offer a powerful therapeutic tool for victims of h‐ARS and potentially for recipients of organ transplants.

## Introduction

A radiological or nuclear disaster may result in exposure to potentially lethal doses of ionizing radiation (IR). Accidental or deliberate exposure to a radiation dose greater than 1 Gy can damage the highly proliferative hematopoietic system, leading to myelosuppression. Quiescent, noncycling hematopoietic stem cells (HSCs) are more resistant to IR and retain their ability to recover with doses up to 6 Gy. At progressively higher doses, IR‐induced DNA damage inhibits HSC self‐renewal capacity, leading to cell cycle arrest, apoptosis, and, ultimately, bone marrow (BM) failure and hematopoietic acute radiation syndrome (h‐ARS). At higher doses of IR exposure, the gastrointestinal tract and central nervous system will also be damaged 1, 2.

h‐ARS will manifest itself with pancytopenia, followed by increased risk for bleeding, infections, and eventual death. Several pharmacologic agents are under evaluation as radiomitigators, including antiapoptotic agents (Toll‐like receptor 5 agonist), antioxidants (tocols, nicaraven), and growth factors (interleukin‐12 and granulocyte colony‐stimulating factor 3, 4, 5, 6, 7. It has been well established that growth factors promote BM recovery, but they are unequivocally insufficient as single agents to restore long‐term hematopoiesis in humans 8, 9, 10. However, at radiation exposure doses of less than 3 Gy, short‐term combination therapy with a pharmacological radiomitigating agent, growth factors, and antibiotics may be beneficial and sufficient 11, 12. In contrast, exposure to higher doses of radiation (>7 Gy) would require hematopoietic cell transplantation (HCT) to restore marrow function or provide transient support during host hematopoiesis recovery, as observed in a small number of radiation victims given matched allogeneic HCT 13.

HCT is a well‐established, extensively validated therapeutic option for the treatment of multiple hematologic malignancies and several nonmalignant diseases. However, in the event of a nuclear or radiological incident, HCT will not be a feasible option for most victims without an available matched sibling and will be complicated by the length of time required to find a suitably matched unrelated donor. Ex vivo expanded hematopoietic stem and progenitor cells (HSPCs) can potentially overcome this problem, especially if they can be administered without the requirement for human leukocyte antigen (HLA) matching. We have developed an ex vivo expansion system using the engineered Notch ligand Delta1 and have shown the ability to markedly increase the number of HSPCs with short‐term lymphoid and myeloid repopulating ability in both mouse and human cord blood (CB) 14, 15. Transplantation of CB‐derived HSPCs expanded ex vivo in the presence of Notch ligand have overcome the significant delay in neutrophil recovery after myeloablative CB transplantation (CBT) 16. This expanded CB HSPC product has now been developed for use as a cryopreserved, universal donor (non‐HLA matched), off‐the‐shelf (OTS) cell therapy that has been tested as part of the donor graft in recipients undergoing myeloablative CBT 17. Furthermore, the potential use of ex vivo generated allogeneic mouse myeloid progenitors and xenogeneic CB mononuclear cells as a radiation countermeasure tool has been shown in mouse models of h‐ARS 18, 19, 20.

Allogeneic HCT promotes donor‐specific immune tolerance and subsequently decreases the risk for acute and chronic graft rejection in recipients of solid organ transplants 21, 22, 23. Successful conveyance of allograft immune tolerance in the nonmyeloablative HSCT setting with persistent mixed chimerism and complete withdrawal of immunosuppressive drugs has already been shown in renal transplant recipients 24, 25, 26. Interestingly, renal allograft tolerance has been induced even with transient chimerism in nonhuman primates and humans 27, 28. Kawai et al. hypothesized that this phenomenon was due to transient expansion of donor hematopoietic cells, such as immature dendritic cells or T cells, which may result in thymic deletion of donor‐reactive recipient T cells or induction of donor‐specific regulatory T cells 28.

Herein, we report that infusion of a mismatched cryopreserved ex vivo expanded mouse HSPC (Lin‐Sca1+cKit+ [LSK] cells) product after a lethal dose of IR results in rapid donor engraftment, mitigates IR‐induced toxicity, and improves survival in a dose‐dependent manner, even after the treatment is delayed for 3 days after exposure to lethal IR. Moreover, these cells induce donor‐specific immune tolerance, resulting in longer survival of donor skin allografts. These data reinforce that this clinically relevant, cryopreserved, universal donor, OTS cell product is a feasible therapeutic option for prophylaxis, mitigation, and treatment of IR‐induced toxicities for victims of h‐ARS and that it may also be a clinically relevant approach to induce donor‐specific tolerance in organ transplant recipients.

## Materials and Methods

### Mice

Female or male B6‐Ly5a (H‐2b, CD45.1+) mice were bred and maintained in the Animal Health Resources center of the Fred Hutchinson Cancer Research Center (FHCRC) under specific pathogen‐free conditions. Female BALB/cJ (H‐2d, CD45.2+) and C3H (H‐2k, CD45.2+) mice were purchased from the Jackson Laboratory (Bar Harbor, ME). Mice were maintained under standard conditions, and all experiments were performed under the approval and guidance of the FHCRC Institutional Animal Care and Use Committee (IACUC).

### Isolation and Expansion of Mouse Hematopoietic Stem and Progenitor Cells

LSK cells from B6‐Ly5a mouse BM were enriched by using the fluorescence‐activated cells sorter (FACS) Aria (Becton Dickinson [BD], Franklin Lakes, NJ, www.bd.com) as previously described 29. After each sort, the purity of the sorted populations was confirmed and exceeded 90%. Nontissue culture‐treated 6‐well plates were coated with engineered Notch ligand (Delta1^ext‐IgG^; DXI) or human IgG at a concentration of 5 µg/ml for 2 hours at 37°C, then washed with phosphate‐buffered saline (PBS) and blocked for at least 30 minutes with PBS containing 2% bovine serum albumin. Sorted LSK cells were cultured in the presence of DXI or IgG in Iscove's modified Dulbecco medium (Thermo Fisher Scientific Life Sciences, Waltham, MA, http://www.thermofisher.com) supplemented with 20% fetal bovine serum (Hyclone FBS, Thermo Fisher Scientific Life Sciences), 1% penicillin‐streptomycin, and the following cytokines: murine stem cell factor, human Flt‐3 ligand, human IL‐6 (100 ng/ml each), and human IL‐11 (10 ng/ml; all cytokines purchased from PeproTech, Rocky Hill, NJ, https://www.peprotech.com) 14. Cell density was maintained at 1 × 10^6^ cells/ml during the 14‐day culture. At the end of 14 days, expanded LSK cells were harvested and fresh cells were used for transplantation experiments or cryopreserved in 90% FBS + 10% dimethyl sulfoxide. On the day of transplantation, post‐thaw cell recovery and preservation of LSK phenotype were determined by using trypan blue dye exclusion and flow cytometry, respectively.

### Irradiation, Hematopoietic Stem Cell Transplantation, and Tracking Donor Chimerism

Female BALB/cJ mice, 6–8 weeks old, received a single dose of 6.5–8.5 Gy γ‐irradiation using a Cesium source (JL Shepherd & Associates, San Fernando, CA, http://www.jlshepherd.com) at a rate of 81.4 cGy/min. Four to 72 hours later, mice were injected intravenously with IgG‐ or DXI‐expanded fresh or cryopreserved LSK cells (1, 3, 5, and 15 × 10^6^ cells as indicated). To omit the effect of sex, avoid confounding variables, and decrease experimental size we used only female mice as recipients for this study. Once we confirmed that IgG‐expanded cells did not result in donor reconstitution, control mice were injected with saline solution in subsequent experiments. Mice were observed daily, and moribund animals that met the specific criteria established by the IACUC‐approved protocol were euthanized and documented as an experimental death due to radiation‐induced toxicity. Donor chimerism (% CD45.1+ cells) and lympho‐myeloid lineage distribution were documented in the peripheral blood (PB) and BM in a separate cohort of mice, by flow cytometry at indicated times following irradiation, as previously described.

### Flow Cytometry

LSK cells from Ly5a mice BM were enriched by using FACS as previously described 29. Briefly, BM cells from B6‐Ly5a mice were incubated with a lineage (LIN) cocktail prepared in house. The LIN cocktail included antibody against CD2 (clone RM2‐5), CD3 (clone 17A2), CD5 (clone 53‐7.3), CD8a (clone 53‐6.7), CD11b (clone M1/70), GR1 (clone RB6‐8), B220 (clone RA3‐6B2), and TER‐119 (clone TER‐119). All antibodies were from BD Biosciences and raised in rats. After 10 minutes' incubation with LIN cocktail, the samples were washed and sheep anti‐rat IgG beads (Dynabeads, ThermoFisher Scientific Life Sciences) were added. The LIN‐positive cells were separated using by DynaMag magnets (ThermoFisher Scientific Life Sciences). LIN‐negative cells were stained with Sca1‐PE (clone E13‐161.7) and c‐kit‐fluorescein isothiocyanate (FITC) (clone 2B8), and LSK cells were isolated using an FACS ARIA II cell sorter.

Blood samples were collected by using the retro‐orbital technique, and BM cells were aspirated from the right or left femur under general anesthesia. Following red cell lysis, PB and BM cells were incubated with a blocking reagent (PBS with 2% FBS, an anti CD16/CD32 antibody (2.4G2), and stained with the following antimouse‐specific antibodies (all from BD unless noted); CD45.1‐PE‐Cy7 (cloneA20), CD45.2‐allophycocyanin (APC)‐Cy7 (clone104), CD3‐FITC (clone 17A2), Gr1‐APC (cloneRB6‐8C5), B220‐APC (cloneRA3‐6B2). Flow cytometric analysis was performed by using LSRII (BD Biosciences). All flow cytometry data were analyzed by using FlowJo software, version 9.0 (TreeStar, CA, http://www.flowjo.com).

### Skin Graft Procedure

In a subset of mice surviving >40 days, donor‐specific tolerance was evaluated by subjecting these mice to bilateral allogeneic and syngeneic skin grafting. Bilateral allogeneic and syngeneic skin grafting was performed in two groups of BALB/cJ mice. The first group received a BALB/cJ skin graft on the left side and either a B6‐Ly5a or a C3H (H‐2k, CD45.2) skin graft on the right side. The second group of BALB/cJ mice received a B6‐Ly5a skin graft on the left and a C3H skin graft on the right side. The technique was adapted from a previously reported method 30. Briefly, donor BALB/cJ, B6‐Ly5a, and C3H mice were euthanized and the ventral and lateral trunk skin was collected, cut into small squares, and kept in cold PBS. Control (reconstituted with BALB/cJ bulk BM cells) and chimeric BALB/cJ mice were anesthetized with isoflurane, 7‐ to 10‐mm graft beds were prepared bilaterally on the dorso‐lateral thorax, the skin graft was placed and trimmed to size in situ, and the corners were anchored with interrupted sutures (5.0 wax‐coated braided silk). Grafts were dressed with nonadherent absorbent gauze pads, paper tape, and vet wrap. After 7 days, the dressings and sutures were removed and the grafts were scored daily thereafter. The day of rejection was defined as >80% of the graft being necrotic, scabbed, or dislodged from the graft bed.

### Statistical Analysis

All statistical analyses were performed by using Prism software, vVersion 6.0f (GraphPad, San Diego, CA, https://www.graphpad.com) and *p* values <.05 were considered to represent statistically significant differences. Results of experiments are represented as the mean ± SEM. Engraftment data were analyzed by using a standardized Student *t* test, and overall survival and graft survival were analyzed by using Kaplan‐Meier survival curve analyses. Logistic regression was used to calculate the dose of radiation expected to cause death to 50% of an exposed population within 30 days and to 70% of an exposed population within 30 days (LD_70/30_). The skin graft survival data were analyzed by using a stratified Wilcoxon (Breslow) test for equality of survivor functions.

## Results

### Infusion of Mismatched Expanded Murine Progenitor Cells After Lethal Radiation Results in Rapid Myeloid Recovery and Improved Survival

**Figure 1:**
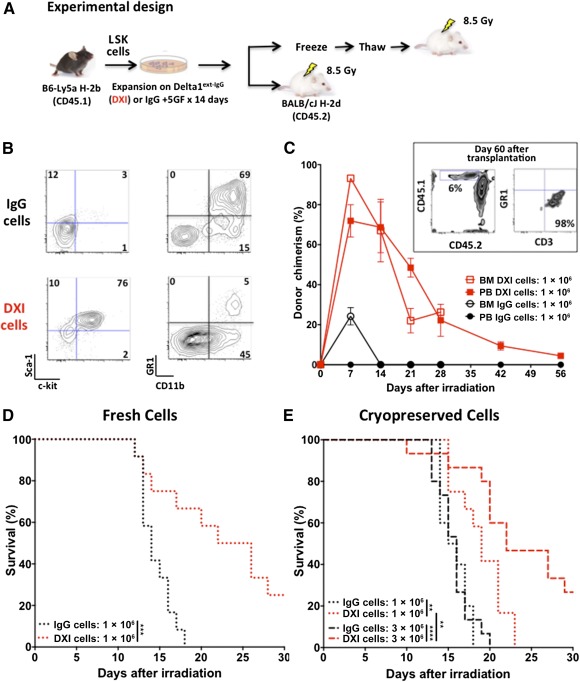
Infusion of DXI‐cultured cells reconstitutes major histocompatibility complex‐mismatched recipients and mitigates total‐body irradiation (TBI)‐induced mortality. **(A):** Experimental design. The LSK from B6‐Ly5a (H‐2b, CD45.1) mice were sorted and cultured on DXI‐ (5 μg/ml) or IgG‐coated flasks for 2 weeks. DXI or IgG‐cultured cells, fresh, at the end of culture, or previously cryopreserved were transplanted intravenously into 6‐ to 8‐week‐old BALB/cJ (H‐2d, CD45.2) mice within 2–4 hours after the mice had been lethally irradiated with 8.5 Gy TBI (^137^Cs γ rays). (**B):** Flow cytometric analysis of the IgG‐ (top panels) and DXI‐cultured (lower panels) cells at the end of 14‐day culture. **(C):** Percentage of donor cells (45.1+) in PB and BM at indicated time points after transplantation of 1 × 10^6^ fresh DXI‐ or IgG‐cultured cells. Inset shows flow cytometric analysis of donor (45.1+) cells (left panel) and myeloid and T‐lymphoid lineage distribution (right panel) of donor cells in PB from a representative mouse transplanted with allogeneic DXI‐cultured cells at 60 days after transplantation. Percentage of donor cells (45.1+) ± standard mean error (bars) in PB and BM. **(D):** Thirty‐day survival rate of mice after transplantation of 1 × 10^6^ fresh DXI‐ or IgG‐cultured cells (*n* = 12 for each group). **(E):** Thirty‐day survival rate of mice after transplantation of 1 or 3 × 10^6^, previously cryopreserved (off‐the‐shelf) DXI‐ and IgG‐cultured cells (*n* = 12 and 10 for 1 × 10^6^ DXI and IgG groups, respectively; *n* = 15 for 3 × 10^6^ DXI and IgG groups). Log‐rank (Mantel‐Cox) test was used to analyze the survival statistics. ∗∗, *p* < .01; ∗∗∗, *p* < .001; ∗∗∗∗, *p* < .0001. Abbreviations: BM, bone marrow; DXI, Delta1^ext‐IgG^; IgG, immunoglobulin G; LSK, Lin‐Sca1+cKit+; PB, peripheral blood; TBI, total body irradiation.

We have previously shown that mouse and human HSPCs expanded in cultures containing fibronectin fragments and immobilized Notch ligand efficiently repopulate syngeneic and xenogenic recipients 14, 15. Herein, we tested whether expanded murine HSPCs could similarly provide rapid hematopoietic reconstitution when infused into MHC mismatched recipients after lethal radiation. To achieve this, we injected fresh 1 × 10^6^ B6‐Ly5a (H‐2b, CD45.1) LSK cells, expanded with IgG or Delta1^ext‐IgG^ for 14 days, into lethally irradiated (8.5 Gy) 6‐ to 8‐week‐old female BALB/cJ (H‐2d, CD45.2) mice (Fig. 1A). As expected, at the end of the 14‐day culture period, 76% of the Delta1^ext‐IgG^‐cultured cells were Sca‐1+ c‐Kit+ (Fig. 1B, left lower panel), and few expressed the granulocyte‐associated (GR‐1 and CD11b) antigens (Fig. 1B, right lower panel). In contrast, few cells cultured with control IgG were Sca‐1+ c‐Kit+, and most were GR‐1+ and CD11b+ granulocytes, indicating differentiation (Fig. 1B, left and right top panels).

As early as 7 days after infusion with fresh DXI‐cultured cells, a high level of engraftment was observed in both PB and BM of MHC mismatched mice (Fig. 1C). In these mice, donor cells continued to decrease over 8 weeks, resulting in a low level of donor cells in PB (4.5% ± 0.6%) up to 60 days after transplant. In contrast, donor engraftment at day 7 was low (24% ± 4%) in mice infused with control IgG‐cultured cells and was detected only in the BM. By day 14, no donor engraftment was detected in this group (Fig. 1C). Early donor‐derived hematopoietic reconstitution with DXI‐cultured cells was predominantly myeloid (data not shown), whereas at 2 months after transplant, the donor‐derived hematopoiesis consisted entirely of T‐lymphoid cells, progeny of short‐term repopulating cells expanded ex vivo (Fig. 1C, inset). Infusion of DXI‐expanded HSPCs, relative to control IgG‐cultured cells, resulted in protection from death after a lethal dose of 8.5 Gy total‐body irradiation (TBI); median survival was 14 days for mice receiving IgG‐cultured cells versus 24 days with DXI‐cultured cells. None of the mice receiving IgG‐cultured cells were alive 30 days after infusion of the cells, whereas 25% in the DXI group were alive at this time point (Fig. 1D).

We next determined whether our expanded HSPCs could be cryopreserved and used as an off‐the‐shelf product to mitigate TBI‐induced mortality after a lethal dose of 8.5 Gy TBI, similar to the expanded cells infused fresh after culture. Treatment with cryopreserved DXI‐cultured cells mitigated TBI‐induced mortality in a cell dose‐dependent manner. None of the mice transplanted with 1 × 10^6^ cryopreserved DXI‐cultured cells survived beyond day 23; however, mortality was significantly delayed compared with that in mice transplanted with IgG‐cultured cells (Fig. 1E). In contrast, overall survival was 27% after transplantation with 3 × 10^6^ DXI‐cultured cryopreserved cells (median survival of 22 days), whereas none of the mice in the IgG group survived beyond day 20 (*p* ≤ .001) (Fig. 1E). Of note, the kinetics of donor cell engraftment in these recipients was similar to that in the mice treated with fresh cells. These results confirmed that infusion of cryopreserved, mismatched DXI‐cultured cells shortly after lethal TBI (within 2–4 hours) mitigated IR‐induced mortality in a cell dose‐dependent manner.

### Treatment With Cryopreserved Allogeneic DXI‐Cultured Cells Improves Survival of Mice Exposed to a Range of Lethal Doses of TBI

**Figure 2:**
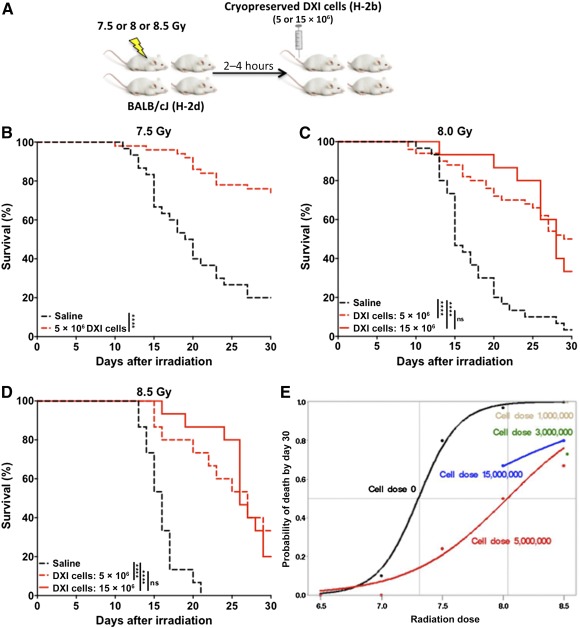
Treatment with cryopreserved allogeneic DXI‐cultured cells shortly after exposure to lethal doses of total‐body irradiation (TBI) improves survival. **(A):** Experimental design. Five or 15 × 10^6^ cryopreserved, DXI‐cultured B6‐Ly5a (H‐2b) cells were injected into BALB/cJ (H‐2d) mice 2–4 hours after mice had been irradiated with 7.5‐, 8.0‐, or 8.5‐Gy TBI (^137^Cs γ rays). Thirty‐day survival rate of mice after injection with **(B)** 5 × 10^6^ cryopreserved DXI‐cultured cells or saline after 7.5‐Gy TBI (*n* = 50 mice in the DXI group and *n* = 30 mice in the vehicle group; 3 independent experiments), **(C)** 5 or 15 × 10^6^ cryopreserved DXI‐cultured cells or saline after 8.0‐Gy TBI (*n* = 50 and *n* = 15 mice in DXI group 5 and 15 million, respectively, and *n* = 30 mice in vehicle group; 3 independent experiments), and **(D)** 5 or 15 × 10^6^ cryopreserved DXI‐cultured cells or saline after 8.5‐Gy TBI (*n* = 15 mice per group; 1 experiment). **(E):** Predicted 30‐day lethality after various doses of radiation in mice treated with saline and 1–15 × 10^6^ cryopreserved DXI‐cultured cells using logistic regression at intervals of 50 rads. The dose of radiation expected to cause death to 50% of an exposed population within 30 days was 7.3 Gy, and the dose of radiation expected to cause death to 70% of an exposed population within 30 days was 7.45 Gy for control BALB/cJ mice. Black dots and line, mice treated with saline; brown dot, mice treated with 13106 DXI‐cultured cells; green dot, 33106 DXI‐cultured cells; red dots and line, 53106 DXI‐cultured cells; blue dots and line, 153106 DXI‐cultured cells. There are no lines for mice treated with 1 or 3 × 10^6^ cells because only one TBI dose was used. ∗, *p* < .05; ∗∗, *p* < .01; ∗∗∗, *p* < .001; ∗∗∗∗, *p* < .0001. Abbreviation: DXI, Delta1^ext‐IgG^.

We next evaluated the ability of our cryopreserved expanded progenitor cell product to provide radioprotection at a range of radiation dose exposures. Mice were exposed to a range of TBI doses and were treated with 5 × 10^6^ cryopreserved allogeneic mismatched DXI‐cultured cells or saline 2–4 hours after TBI (Fig. 2A). At a 6.5‐Gy TBI dose, all mice survived in both groups; at a 7.0‐Gy TBI dose, only 1 mouse died at day 28. Therefore, there was no statistical difference between the two groups (data not shown). However, at a single TBI dose of 7.5–8.5 Gy, treatment with DXI‐cultured cells dramatically improved the 30‐day survival rate relative to the control group (Fig. 2B–2D). Surprisingly, treatment with threefold higher cell dose (15 × 10^6^ DXI‐cultured cells) failed to provide any additional survival benefit to mice after 8.0‐ and 8.5‐Gy TBI (Fig. 2C, 2D). Using logistic regression, we showed that the calculated LD_70/30_ dose reduction factor (LD_70/30_ for the treated mice/LD_70/30_ for the control mice) was 1.12 when mice were treated with 5 × 10^6^ DXI‐cultured cells and 1.09 when 15 × 10^6^ DXI‐cultured cells were infused; this finding confirmed that treatment with higher cell dose did not provide additional survival benefit to these mice (Fig. 2E). Taken together, these results confirm that 3–5 × 10^6^ cryopreserved mismatched allogeneic DXI‐cultured cells administered within 4 hours of exposure to lethal TBI is sufficient to mitigate radiation‐induced toxicity.

### Delayed Treatment With Cryopreserved Allogeneic DXI‐Cultured Cells Conveys Survival Benefit After Lethal Doses of TBI

**Figure 3:**
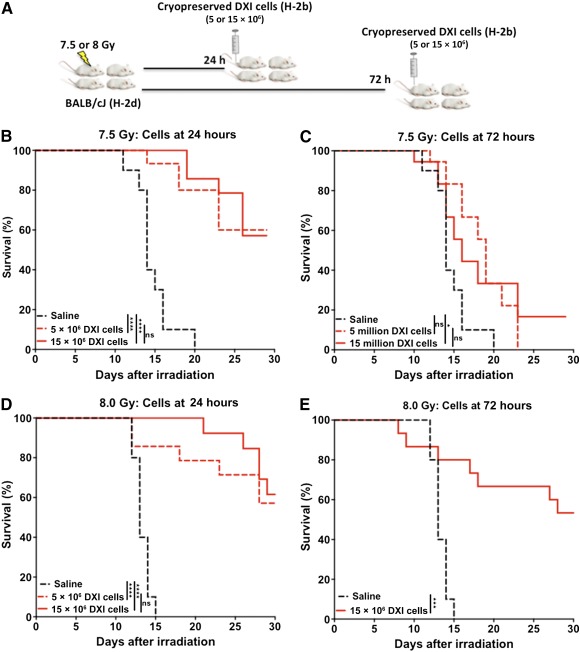
Delayed treatment with cryopreserved allogeneic DXI‐cultured cells improves survival after lethal doses of TBI. **(A):** Experimental design. Five or 15 × 10^6^ cryopreserved, DXI‐cultured B6‐Ly5a (H‐2b) cells were injected into BALB/cJ (H‐2d) mice at 24 or 72 hours after 7.5‐ or 8.0‐Gy TBI. **(B):** Thirty‐day survival rate of mice after injection with 5 or 15 × 10^6^ cryopreserved DXI‐cultured cells or saline at 24 hours after 7.5‐Gy TBI (*n* = 15 and 14 mice in DXI group and *n* = 10 mice in vehicle group). **(C):** Thirty‐day survival rate of mice after injection with 5 or 15 × 10^6^ cryopreserved DXI‐cultured cells or saline at 72 hours after 8.0‐Gy TBI (*n* = 18 mice in both DXI groups and *n* = 10 mice in vehicle group). **(D):** Thirty‐day survival rate of mice after injection with 5 or 15 × 10^6^ cryopreserved DXI‐cultured cells or saline at 24 hours after 8.0‐Gy TBI (*n* = 14 and 13 mice in DXI groups and *n* = 10 mice in vehicle group). **(E):** Thirty‐day survival rate of mice following injection with 15 × 10^6^ cryopreserved DXI‐cultured cells or saline at 72 hours after 8.0‐Gy TBI (*n* = 15 in DXI group and *n* = 10 mice in vehicle group). ∗, *p* < .05; ∗∗, *p* < .01; ∗∗∗, *p* < .001; ∗∗∗∗, *p* < .0001. Abbreviations: DXI, Delta1^ext‐IgG^; TBI, total body irradiation.

Victims of acute radiation‐induced toxicity may not have immediate access to readily available treatment. To test whether delayed infusion of DXI‐cultured cells for 24 or 72 hours after irradiation could still provide radioprotective efficacy, mice were exposed to a TBI dose of 7.5 or 8.0 Gy and treated with DXI‐cultured cells 24 or 72 hours after irradiation (Fig. 3A). Delayed infusion to 24 hours after radiation exposure was again associated with significantly improved 30‐day survival for both the 7.5‐ and 8‐Gy TBI dose groups. None of the mice in the vehicle arms survived; however, up to 60% of the mice treated with DXI‐cultured cells survived the radiation injury at both TBI doses (Fig. 3B, 3D). Once again, survival rate did not further improve by increasing the treatment cell dose (Fig. 3B, 3D). When treatment was delayed for 72 hours, all of the mice treated with 5 × 10^6^ DXI‐cultured cells died of radiation‐induced toxicity after 7.5‐Gy TBI. However, when mice were treated with the increased cell dose of 15 × 10^6^ DXI‐cultured cells, 17% were still alive at 30 days (Fig. 3C). Finally, delayed infusion of the higher cell dose (15 × 10^6^ DXI‐cultured cells) resulted in improved survival after a more toxic 8.0‐Gy TBI dose; 50% survived to 30 days after TBI (Fig. 3E). Collectively, these results confirm that delayed treatment with DXI‐cultured cells mitigates TBI‐induced mortality and that increased cell numbers improve survival when treatment is further delayed.

### Infusion of Mismatched DXI‐Cultured Cells Induces Donor‐Specific Tolerance and Improves Skin Graft Survival

**Figure 4:**
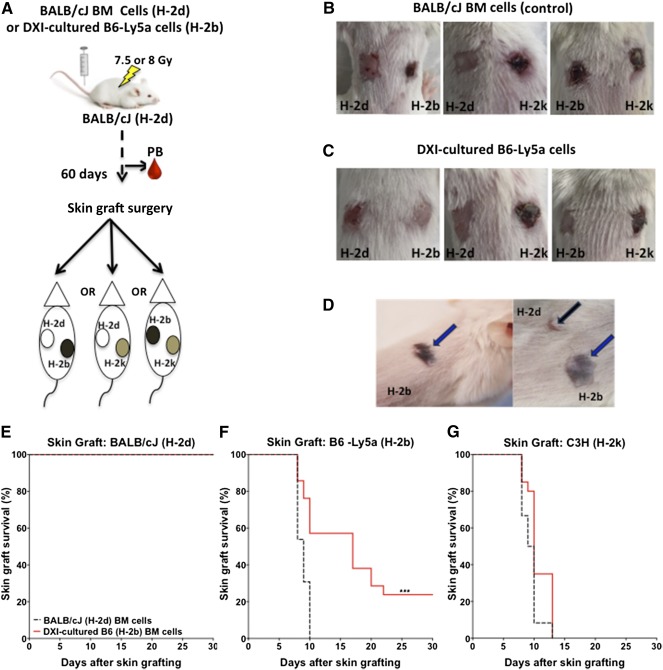
Infusion of cryopreserved allogeneic DXI‐cultured cells induces donor‐specific immune tolerance and improves skin graft survival. **(A):** Experimental design. BALB/cJ (H‐2d) mice were transplanted with 5 × 10^6^ syngeneic BALB/cJ (H‐2d) BM cells (control mice, *n* = 20) or cryopreserved allogeneic DXI‐cultured B6‐Ly5a (H‐2b) cells (DXI mice, *n* = 33) within 2–4 hours after 7.5‐ or 8.0‐Gy TBI. **(B, C**): Sixty days after transplantation, each mouse received 2 skin grafts. Control mice had syngeneic H‐2d (*n* = 9), allogeneic H‐2b (*n* = 13), or third‐party H‐2k (*n* = 12) skin grafts. DXI mice had syngeneic H‐2d (*n* = 21), allogeneic H‐2b (*n* = 21), or third‐party H‐2k (*n* = 20) skin grafts. **(B):** Representative skin grafts in BALB/cJ (H‐2d) mice transplanted with syngeneic BM cells with H‐2d and H‐2b or H‐2d and H‐2k, or H‐2b and H‐2k skin grafts. **(C):** Representative skin grafts in BALB/cJ (H‐2d) mice transplanted with allogeneic DXI‐cultured cells with H‐2d and H‐2b or H‐2d and H‐2k, or H‐2b and H‐2k skin grafts. **(D):** Representative healthy H‐2b (blue arrow) and H‐2d (black arrow) skin grafts in BALB/cJ mice transplanted with DXI‐cultured cells 60 days after surgery. **(E–G):** Thirty‐day skin graft survival rate of syngeneic (H‐2d) (9 in control and 21 in DXI mice) **(E)**, allogeneic (H‐2b) (13 in control and 21 in DXI mice) **(F)**, and third‐party (H‐2k) (12 in control and 20 DXI mice) **(G)** skin grafts. ∗∗∗, *p* < .001. Abbreviations: BM, bone marrow; DXI, Delta1^ext‐IgG^.

Long‐term persistence of low levels of donor T cells in the PB of mice transplanted with DXI‐cultured cells, with no evidence of graft‐versus‐host disease (GVHD), suggested the presence of donor‐specific transplantation tolerance across full MHC barriers. To address whether these mice had developed donor‐specific tolerance, they were challenged by surgical placement of a syngeneic (BALB/cJ, H‐2d), donor (B6‐Ly5a, H‐2b), or third‐party (C3H, H‐2k) skin graft 60 days after they had been transplanted with control syngeneic BM or DXI‐cultured cells. Every mouse was implanted with two skin grafts, one on each side of the flank; the origin of the graft on each flank was syngeneic/donor, syngeneic/third party or donor/third party (Fig. 4A). Six and four graft failures resulting from technical problems occurred in the control and DXI groups, respectively. None of the syngeneic skin grafts were rejected in mice previously transplanted with syngeneic BM (Fig. 4B, 4E) or allogeneic DXI‐cultured cells (Fig. 4C, 4E), whereas all third‐party skin grafts were rejected in all mice within the first 13 days after the graft placement, leaving behind contracted scar tissue (Fig. 4B, 4C, 4G).

In contrast, the 30‐day survival rate of donor grafts was significantly prolonged in the DXI group; by day 30, 48% (10 of 21) of the skin grafts appeared healthy and showed no signs of rejection (crusting and scarring) (Fig. 4C, 4F; *p* ≤ .001). Moreover, 14% of these grafts showed complete engraftment, with evidence of black hair growth on a white hair background at the surgical site (Fig. 4D). Prolonged graft survival in these mice was not due to immune deficiency because the mice rejected all third‐party skin grafts (Fig. 4C, 4G). In stark contrast, all donor grafts were rejected in mice transplanted with syngeneic BM cells (Fig. 4B, 4F). Intriguingly, the level of persistent donor engraftment in the PB at the time of skin grafting did not correlate with graft survival. These results support the view that improved skin graft survival resulted from induction of donor‐specific immune tolerance by infusion of cryopreserved allogeneic DXI‐cultured cells. None of the mice surviving beyond the initial 30 days after TBI developed any long‐term complications of radiation exposure during the experiment (90 days).

## Discussion

Despite substantial efforts in the field, no effective therapy has been developed to mitigate h‐ARS 31. There is an urgent need to develop therapeutic modalities that could bridge the acute immune suppression and myelosuppression in most victims, who may need only more effective supportive care while waiting autologous hematopoietic recovery.

To address this unmet medical need, we investigated the use of ex vivo expanded progenitor cells as a novel therapy for treatment of h‐ARS in a murine model. This approach was based on the previous demonstration that infusion of Notch‐expanded CB HSPCs in the myeloablative CBT setting resulted in a dramatic reduction in median time to neutrophil recovery, from 25 to 11 days 16. However, to be feasible in the treatment of h‐ARS, the ideal therapy would have to be readily available or available on demand and be easily administered. Thus, we developed this product as a non‐HLA‐matched product that could be manufactured and then cryopreserved for future on‐demand use as an off‐the‐shelf product. Herein, we demonstrate that treatment with cryopreserved, ex vivo DXI‐expanded murine HSPCs in MHC mismatched murine recipients after a wide range of lethal TBI doses led to improved overall survival. The observed rapid recovery of donor‐derived myeloid cells by day 7 after the infusion of the expanded cell product, despite the major mismatch between the cells and the recipients, likely contributed to the significant reduction in death among these mice by reducing the risk for infectious complications and other toxicities known to be associated with exposure to high‐dose radiation.

We observed that a larger cell dose was needed to rescue irradiated mice when cryopreserved DXI‐expanded murine LSK cells were transplanted instead of freshly cultured LSK cells. This may have been due to the cryopreservation process, which can reduce cell viability by several mechanisms. It may also reflect the overestimation of viable cells by trypan blue staining, a method that falls short of distinguishing apoptotic cells after thawing. Further studies could test a better method to evaluate viability of the cells after thawing and compare different methods of cryopreservation. Advances in cryopreservation technology and a better assessment of viability of the cells should allow for more accurate dosing with cryopreserved expanded HSPCs.

We also demonstrate that sustained mixed donor chimerism in the BM and PB is possible across major H2 histocompatibility barriers in fully mismatched mice without any evidence of GVHD. Importantly, this study did not use post‐transplant immunosuppression or anti‐host antibody therapy, which was found to be required in previous studies 32, 33, 34. Furthermore, an important feature of h‐ARS treatment is the ability to delay treatment up to 3–5 days after radiation exposure. Delayed infusion of our mismatched, cryopreserved expanded HSPC product up to 3 days after lethal radiation exposure also resulted in a 30‐day survival benefit.

In our murine model of h‐ARS, donor‐derived engraftment peaked at day 7 with a predominance of myeloid cells. Thereafter, the level of donor engraftment declined, and by day 60, donor engraftment stabilized at a low level that was almost exclusively derived of predominantly CD3^+^ T lymphocytes without any evidence of GVHD. T lymphoid donor chimerism in these mice transplanted with MHC mismatched cells is similar to what we previously reported in mice transplanted with MHC matched, DXI‐expanded cells 14, and the cells are the progeny of short‐term lymphoid myeloid repopulating cells generated ex vivo. We do not believe there is an impact of Notch signaling on development of progeny T‐cells generated from expanded repopulating cells because the Notch ligand Delta1 was used to induce proliferation while inhibiting differentiation of LSKs.

The induction of immune tolerance across full MHC barriers was demonstrated by a significantly higher skin graft survival rate in mice transplanted with DXI‐expanded cells. We did not observe any correlation between the level of chimerism and graft survival, as previously reported by others 26, 35. A low level of T‐cell chimerism (3.4% ± 5%; range, 0.2%–11.2%) was sufficient to convey donor‐specific immunological tolerance to the skin grafts. However, the graft survival rate was higher (40%) in mice exposed to 8.0‐Gy TBI than in mice exposed to 7.5‐Gy TBI, in which only 1 of 10 skin grafts was not rejected at day 30. These observations suggest that the acceptance of skin grafts observed here may be as dependent on the level of immunosuppression provided by high‐dose radiation as on the level of T‐cell chimerism. We documented that prolonged skin allograft survival in these mice was specifically due to the recipients’ lack of responsiveness against specific donor antigens by showing that they were responsive against third‐party (C3H) antigens and rapidly rejected third‐party skin grafts. We did not evaluate the mechanism of donor‐specific tolerance established in our model, but previous studies in mice and nonhuman primates suggest that both T‐cell clonal deletion and regulatory mechanisms (by inhibiting donor‐specific alloreactivity) play a critical role in the induction and maintenance of donor‐specific immunological tolerance 36.

The induction of donor‐specific tolerance by establishing mixed chimerism after HSCT has been demonstrated by several groups in animal models and more recently in clinical trials 37, 38. The current study is the first to show the induction of donor‐specific tolerance in recipients treated with cryopreserved, ex vivo expanded allogeneic, and non‐HLA‐matched HSPCs. Similar to these findings of donor‐specific tolerance induction in our murine model, a reduction in the incidence of severe acute GVHD was observed in a small group (*n* = 15) of patients who received off‐the‐shelf, cryopreserved expanded CB HSPCs as part of a nonrandomized pilot study in the setting of myeloablative CBT. A randomized clinical trial is underway in the myeloablative CBT setting to confirm the promising clinical outcomes observed to date with this expanded cell product: faster neutrophil and platelet recovery and the reduction in infectious complications, transplant‐related mortality, and high‐grade GVHD 17.

## Conclusion

We have presented evidence that infusion of cryopreserved, allogeneic ex vivo expanded HSPCs in mice after lethal radiation reduced the risk for death and conveyed donor‐specific immune tolerance in skin allograft recipients. These findings demonstrate that the DXI‐expanded off‐the‐shelf cell product is promising as a therapeutic option to mitigate the risks for prolonged neutropenia and the associated increased morbidity and mortality. Furthermore, this study provides strong evidence regarding the induction of tolerance conveyed by this product in our murine model of h‐ARS. This product will undergo continued development as a treatment for patients who are at risk for prolonged neutropenia as well as for use as a medical countermeasure for h‐ARS and conveying donor‐specific immune tolerance in transplant recipients.

## Author Contributions

F. Milano, F. Merriam: conception and design, collection and/or assembly of data, data analysis and interpretation, manuscript writing; I.N.: collection and/or assembly of data, data analysis and interpretation; J.L.: collection and/or assembly of data; T.A.G. and S.H.: data analysis and interpretation; S.I.: data analysis and interpretation, manuscript writing, final approval of manuscript; C.D.: conception and design, data analysis and interpretation, manuscript writing, final approval of manuscript.

## Disclosure of Potential Conflicts of Interest

S.H. is a compensated consultant for and has stock options in Nohla Therapeutics and serves as a consultant and chair of the SAB. C.D. is chief medical officer, an inventor on patents, a consultant, and a member of SAB/CAB, and has a sponsored research agreement and ownership interest. The other authors indicated no potential conflicts of interest.
